# Application of a Novel Proteomic Microarray Reveals High Exposure to Diarrhoeagenic *Escherichia coli* among Children in Zambia Participating in a Phase I Clinical Trial

**DOI:** 10.3390/microorganisms12030420

**Published:** 2024-02-20

**Authors:** Kapambwe Mwape, Cynthia Mubanga, Obvious Nchimunya Chilyabanyama, Kennedy Chibesa, Caroline Cleopatra Chisenga, Suwilanji Silwamba, Arlo Randall, Xiaowu Liang, Tobias George Barnard, Michelo Simuyandi, Roma Chilengi

**Affiliations:** 1 Enteric Disease and Vaccines Research Unit, Centre for Infectious Disease Research in Zambia, Lusaka P.O. Box 34681, Zambia; kapambwe.mwape@cidrz.org (K.M.); cynthia.mubanga@cidrz.org (C.M.); chilyabanyama@gmail.com (O.N.C.); kennedy.chibesa@cidrz.org (K.C.); caroline.chisenga@cidrz.org (C.C.C.); suwilanji.silwamba@cidrz.org (S.S.); michelo.simuyandi@cidrz.org (M.S.); 2 Water and Health Research Center, Faculty of Health Sciences, University of Johannesburg, P.O. Box 17011, Doornfontein, Johannesburg 2028, South Africa; tgbarnard@uj.ac.za; 3 Department of Basic Medical Sciences, Michael Chilufya Sata School of Medicine, Copperbelt University, Ndola P.O. Box 71191, Zambia; 4 Division of Medical Microbiology, Department of Pathology, Stellenbosch University & National Health Laboratory Service, Tygerberg Hospital Francie van Zijl Drive, P.O. Box 241, Cape Town 8000, South Africa; 5 Next Generation Sequencing Unit and Division of Virology, School of Pathology, Faculty of Health Sciences, University of the Free State, P.O. Box 339, Bloemfontein 9300, South Africa; 6 Antigen Discovery Inc., 1 Technology Dr., STE E309, Irvine, CA 92618, USA; arandall@antigendiscovery.com (A.R.); xliang@antigendiscovery.com (X.L.)

**Keywords:** microarray, vaccines, diarrhoeagenic *E. coli*, diarrhoea, cross-reactivity, ETVAX vaccine

## Abstract

Diarrhoeagenic *E. coli* (DEC) significantly contributes to the burden of diarrhoea among children. Currently, there is no approved vaccine against DEC, but several vaccines against the enterotoxigenic *E. coli* (ETEC) pathotype are in advanced clinical trial stages, including the ETVAX^®^ vaccine, undergoing evaluation in Zambia. This study reports on the reactivity of antibodies from ETVAX^®^ vaccine and placebo recipients in a phase I clinical trial to proteins derived from (DEC) other than ETEC. Plasma samples collected at two time points (prior to any vaccination and post-third dose vaccination) from 16 vaccinated and 4 placebo participants in a phase 1 clinical trial examining the safety, tolerability, and immunogenicity of ETVAX^®^ with dmLT adjuvant were evaluated for IgG response to *E. coli* antigens other than ETEC using the Pan-DEC protein microarray. This was the first field application of the novel pan-DEC array as a new tool in assessing the antigenic breadth of antibody responses induced by the ETVAX vaccine, as well as to assess early life exposure to DEC pathotypes and other bacterial enteric pathogens. We observed that plasma obtained from ETVAX^®^ and placebo recipients had high antibody reactivity to Ipa, SseC and EspB proteins. These findings suggest that there is high exposure early in life to DEC pathogens, like EPEC, EHEC, EAEC and EIEC in addition to ETEC, in the Zambian population. These immunological observations are consistent with the results of recent epidemiological studies assessing the etiology of diarrheal disease among infants and young children in Zambia.

## 1. Introduction

Diarrhoea is one of the leading causes of morbidity and mortality globally posing a major public health threat to children as they are the most affected [1]. The developing world bears most of the global burden of diarrhoeal disease mainly due to limited safe water supply and poor sanitation [2]. Other than the immediate high morbidity and mortality, diarrhoea is associated with impaired cognitive development and malnutrition among children in the longer term [3]. Diarrhoea may be caused by a wide range of pathogens including viruses, protozoa and bacteria. *Escherichia coli* has been reported to be among the top bacterial aetiological agents of diarrhoea among children under the age of five [4,5,6,7,8]. *E. coli* generally exist as commensals of the gut, but within these species are pathogenic strains that are referred to as diarrhoeagenic *E. coli* (DEC) [9]. Diarrhoeagenic *E. coli* has been classified into six pathotypes based on the virulence genes carried by the organism. These include enterotoxigenic *E. coli* (ETEC), enteropathogenic *E. coli* (EPEC), enteroaggregative *E. coli* (EAEC), enterohaemorrhagic *E. coli* (EHEC) and enteroinvasive *E. coli* (EIEC) [10]. The diagnosis of these pathotypes is not routinely performed as it requires the use of molecular methods which are expensive and not readily available in resource-limited settings [11]. Although studies have been limited when assessing DEC, particularly EPEC, EAEC and EIEC, these pathotypes have been shown to be important contributors to both acute illness and the more long-term sequelae associated with a high burden of enteric infection early in life among infants and young children in LMIC [6,7,12]. An even less studied area is the extent to which DEC pathotypes share antigens and virulence factors, although there is some evidence to indicate they can [12]. This potential for sharing virulence factors and antigens has vaccine development implications since it could impact on the breadth of vaccine coverage. Consequently, more research is needed in this area as it may be an important factor that could contribute to the ETEC and DEC vaccine value proposition [6].

The management of bacterial diarrhoea requires the use of antibiotics as they are effective in arresting bacterial multiplication and therefore limit shedding and the duration of the illness [13]. However, the indiscriminate use of antibiotics has contributed to the emergence and spread of antibiotic-resistant strains, which has resulted in treatment challenges. The multi-drug-resistant strains of DEC have been reported in the sub-Saharan region [14,15,16]. Hence, prevention rather than the treatment of severe diarrhoea is the scientifically preferable option for combating diarrhoeal diseases. While water, sanitation and hygiene (WASH) interventions are the mainstay for the prevention and control of diarrhoea, it is a far-fetched future solution in low- or middle-income countries (LMICs) due to the huge implementation costs [17]. Therefore, the use of vaccines could provide a crucial solution to this problem. Currently, no vaccine against any of the DEC pathotypes is approved for clinical use in humans. However, substantial progress has been made towards the development of a vaccine against ETEC, which is an important cause of travelers’ diarrhoea and watery diarrhoea among children under five years in the developing world [18]. Enterotoxigenic *E. coli* vaccines in the most advanced stages of development include the inactivated whole cell vaccine, ETVAX^®^, live attenuated combination candidates like ShigETEC and 1208S-122 and subunit approached, purified and fimbrial tip adhesins with the new dmLT (double mutant heat-labile toxin) adjuvant [6,18,19,20]. These vaccines target the colonisation factors and/or (heat-labile) LT toxins of ETEC [21]. There is, however, no multivalent vaccine to cover the various DEC pathotypes, although as indicated above there is evidence that ETEC and some other DEC strains may share antigens [6,12]. Therefore, information on the scope of protection offered by the ETVAX^®^ vaccine beyond the ETEC pathotype could be scientifically relevant in high diarrhoeal disease burden areas. The ETVAX^®^ vaccine comprises four *E. coli* strains, each overexpressing the colonization factors CFA/I, CS3, CS5 and CS6 combined with LCTBA protein (a fusion of the binding subunits found in both ETEC heat-labile toxin (LTB) and cholera toxin (CTB) [22]. Cross-reactive antibodies against the antigens of the same species occur when there are conserved epitope regions across species; genetic information coding for virulence proteins and other antigenic factors can also be shared across pathotypes [23]. Hence, a whole cell vaccine designed against one of the DEC pathotypes may induce a cross-reactive response to other pathotypes, and thereby potentially confer broader clinical protection. The pan-DEC arrays being evaluated for the first time in this study may be a valuable new tool in this regard since it enables vaccine developers to look more broadly at antibody levels against DEC antigens in endemic populations as well as those that may be inducted by vaccine candidates in development, like ETVAX.

Using the new pan-DEC microarray platform, this study sought to identify DEC antigens to which antibodies obtained from the phase I clinical trial participants may react beyond the ETEC pathotype via immune-proteomic profiling. Microarrays have previously been used to determine the cross-reactivity of antibodies to organisms belonging to the same genus or species [24,25,26]. This platform provides a large spectrum of proteins that can be probed at once while maintaining great specificity and detail [27].

## 2. Materials and Methods

### 2.1. Summary of the Parent Study and Selection of Samples for the Sub-Analysis

This was a sub-analysis that emanated from a single-site, double-blind, placebo-controlled, age-descending phase 1 clinical trial assessing the safety, tolerability and immunogenicity of an oral inactivated Enterotoxigenic *Escherichia coli* (ETEC) Vaccine (ETVAX^®^) containing dmLT adjuvant [28]. The principal study comprised three demographic cohorts: 40 adults 18–45 years (cohort A), 60 children aged 10–23 months (cohort B) and 146 children aged 6–9 months (cohort C) [28]. Cohort A received either a placebo or full vaccine dose while the other two cohorts received either a placebo, ¼ or 18th of the adult dose. Each participant received three oral doses of the vaccine over a 90-day period on the first day of the study, day 15 and day 90. The full dose consisted of 150 mL of 1 × effervescent buffer, 8 × 10^10^ inactivated bacterial cells and 1 mg of LCTBA and 10 µg of dmLT. To reconstitute the vaccine, a ¼ or 18th dose of whole cell vaccine and LCTBA was added to 10 mL (at 2 × times the strength of the adult dose), to which 2.5 µg of dmLT was added. For placebo recipients, only 10 mL of effervescent buffer was given. Blood samples for analysis were collected at three time points: pre-vaccination, and 7 days post the second and third dose.

A total of 20 out of 60 randomly selected samples from in cohort B with an age range of 10–23 months were included in this study, representing both vaccinated groups (at two different doses; ¼ and 18th dose) and placebo arms. The 20 samples comprised 4 samples from the placebo and 16 from the vaccinated arm (8 received the ¼ dose, and the other 8 received the 18th dose). Blood samples collected before any vaccination (pre-vaccination) and seven days after the third dose were selected and sent to Antigen Discovery Incorporated (ADI) in the USA for microarray analysis.

### 2.2. Ethical Considerations

The study received ethical approval from the University of Zambia Biomedical Research Ethics Committee (UNZABREC), with the clinical trial registered in the Pan African Clinical Trials Registry (PACTR) under trial number OEV-124 PACTR201905764389804. The protocol underwent review and authorization by the National Health Research Authority (NHRA) and the Zambia Medicines Regulatory Authority (ZAMRA). Written informed consent was obtained from parents/guardians before participant involvement.

### 2.3. Laboratory Analysis

#### Microarray Creation

The Pan-DEC microarray was created by ADI as previously described [29] and represents a new analytical tool that complements an earlier array focused on ETEC [30] comprising 800 potential surface proteins and an additional 4168 gene features. These features were identified through a comparative analysis of 207 clinical ETEC isolates. The selected ETEC genes were found in more than 40% of the ETEC isolates and were absent in the genomes of three common *E. coli* commensal strains. Similarly, following a comparative genomic analysis of non-ETEC pathogenic *E. coli* species, 2168 protein-coding genes were chosen to represent EHEC (730 features), EIEC (452 features), EPEC (456 features), EAEC (288 features) and ExPEC (242 features). These genes were specific to each species, not found in commensal *E. coli*, and were not previously represented in the described ETEC proteome chip. To specifically focus on DEC, the analysis of ExPEC gene features was excluded in the final analysis. The controls included in the DEC microarray consisted of blank IVTT reactions serving as negative controls for protein expression, IgG and IgA antibodies for secondary antibody quality control and blank buffers. These selected genomic features were expressed into proteins using a cell-free in vitro transcription–translation (IVTT) system with each of the proteins containing a 5′ polyhistidine (HIS) epitope and a 3′ haemaglutinin (HA) epitope. These proteins were then printed onto nitrocellulose-coated glass slides using a robotic printer and validated before utilization.

### 2.4. Sample Analysis

Test plasma samples and controls were applied to the microarray and incubated, with the subsequent antibody-antigen reaction quantified using the GenePix 4300 Microarray Scanner (Molecular devices, Sunnyvale, CA, USA). Following scanning and quantification, automated data extraction and quality control were performed in R(R Core Team, 2017). The median local background fluorescence intensity was subtracted from the median fluorescence intensity of the spot foreground to obtain raw signals. Furthermore, normalized signals were derived through the computation of the ratio between the raw signal of a spot and the sample-specific median of IVTT control spot signals, followed by the application of a base-2 logarithmic transformation. For purified recombinant proteins and peptide libraries lacking background signals from the IVTT system, the raw signals underwent a base-2 logarithmic transformation. Both raw and normalized signals underwent specific transformations, while quality control metrics were utilized to detect irregularities or anomalies within the arrays [26].

### 2.5. Data Analysis

The IVTT-expressed protein data underwent a refinement process in which reactive antigens were identified, and non-reactive spots were excluded from further analysis (however, raw and normalized data for all spots were retained). Reactivity filtering involved defining seropositivity as a normalized signal equal to or greater than 1.0, equating to double the sample-specific median IVTT control spot signal and denoting the background level. Antigens were classified as reactive and subjected to subsequent statistical analysis if they exhibited seropositivity in at least 10% of samples from one or more participant groups. It is important to note that raw and normalized data for all array spots were preserved. For purified protein and LPS array spots, normalization exclusively involved the application of the base-2 logarithmic transformation. No specific reactivity subset was applied to the purified protein and LPS data; instead, all spots were included for statistical analysis. To comprehensively illustrate the microarray responses across all participant groups, proteins displaying the highest signal intensity 7 days following the third dose were chosen. The difference in signal intensities between samples obtained pre-vaccination and post full vaccination was utilized to ascertain the delta increase in signal reactivity.

## 3. Results

### 3.1. Selection of Clinical Samples for the Microarray

All plasma samples from the 20 participants (10–23 months) were screened for IgG reactivity to DEC pathotypes EHEC, EPEC, EIEC and EAEC proteins. Participant characteristics are described in Table S1. Furthermore, the various proteins used in the proteomeic microarray are outlined in Supplementary Table S2.

### 3.2. Top 20 Reactive Proteins

**Figure 1:**
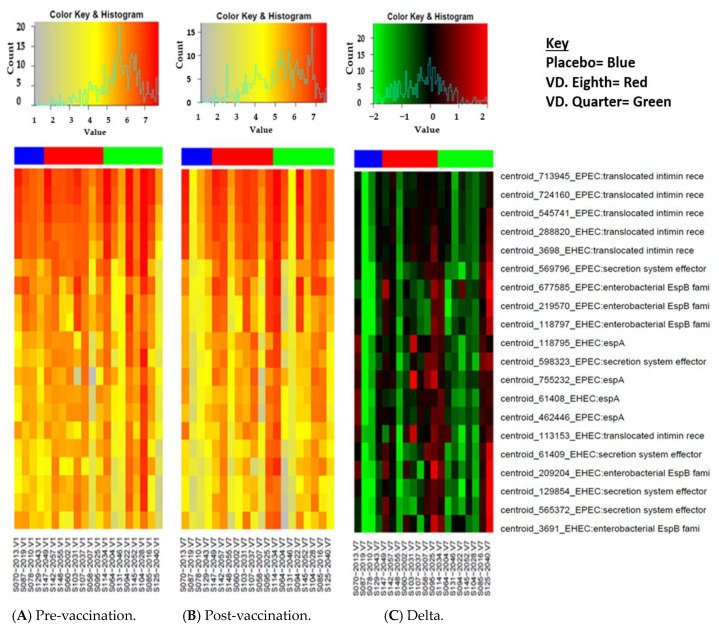
Heatmap of the top 20 reactive proteins by an average of all samples. The proteins are arranged in descending order with the most reactive being at the top. The red indicates the highest signal intensity while the grey indicates the lowest. The blue bar at the top represents the placebo group, the red bar represents the participants that received the 18 dose and the green bar represents participants that received the ¼ dose. Panel (**A**) shows the intensity of reactivity re-vaccination, panel (**B**) shows the intensity of reactivity 7 days post the third dose of vaccination and panel (**C**) shows the difference (delta changes) in the reactivity between pre and post the third dose of vaccination. The different colour illustrates the intensity of the reactivity.

We observed that there was generally a high reactivity across all three groups at both pre- and post-vaccination samples to the top 20 reactive proteins (Figure 1A–C). The delta changes revealed that the intensity of reactivity was generally maintained between the two vaccination time points. Enteropathogenic *E. coli* and EHEC proteins were the only pathotypes in the top 20 reactive proteins across all samples with EPEC proteins (translocated intimin receptor) making up the top 3.

### 3.3. Top 10 Reactive Proteins by Mean Differences in the Vaccinated Group

**Figure 2:**
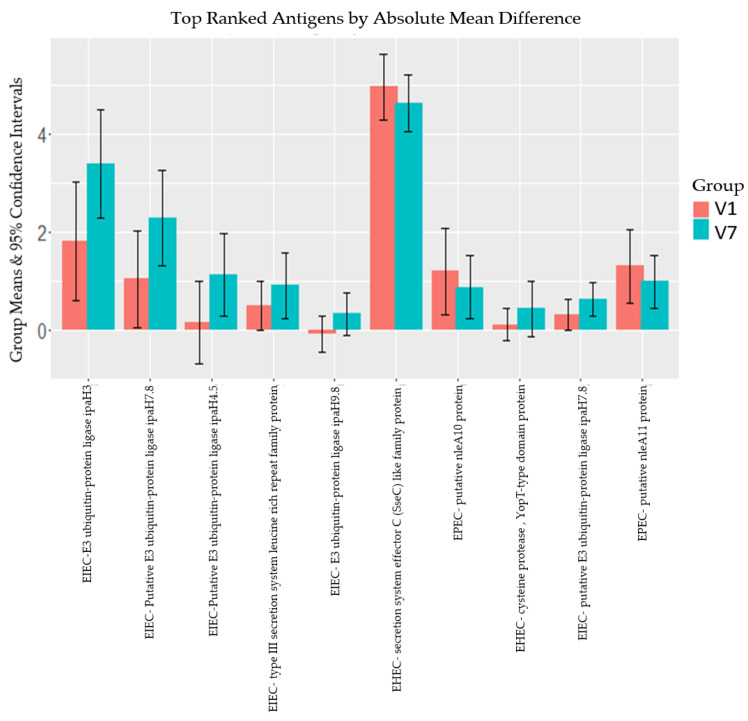
Antibody mean difference in the top 10 reactive proteins among vaccinated individuals between V1 and V7 (D0 and D97). The red bars represent the group mean intensities obtained pre-vaccination (V1) while the blue bars represent the group mean intensities obtained 7 days post the third dose (V7) of the vaccine. The vertical error bars define the 95% confidence interval around each mean. The descriptions of the proteins are in Supplementary Table S1.

When we compared the average signal intensities of the pre-vaccination samples (V1) and the signal intensities of samples obtained 7 days post the last dose (V7), we observed that IgG antibody means were generally higher at 7 days post the third dose of vaccination than at baseline. This was the case for 7 proteins out of the top 10 proteins represented here, except for the centroid EHEC secretion system effector C-like family, EPEC putative nleA10 and EPEC putative nleA11 proteins (Figure 2). There was an increase in IgG for EIEC proteins E3 ubiquitin–protein ligase ipaH3, putative E3 ubiquitin–protein ligase ipaH7.8, putative E3 ubiquitin–protein ligase ipaH4.5, E3 ubiquitin–protein ligase ipaH9.8 and putative E3 ubiquitin–protein ligase ipaH7.8 (*p*-value ≥ 0.05).

### 3.4. Top 10 Reactive Proteins by Antibody Mean Differences in the Placebo Group

**Figure 3:**
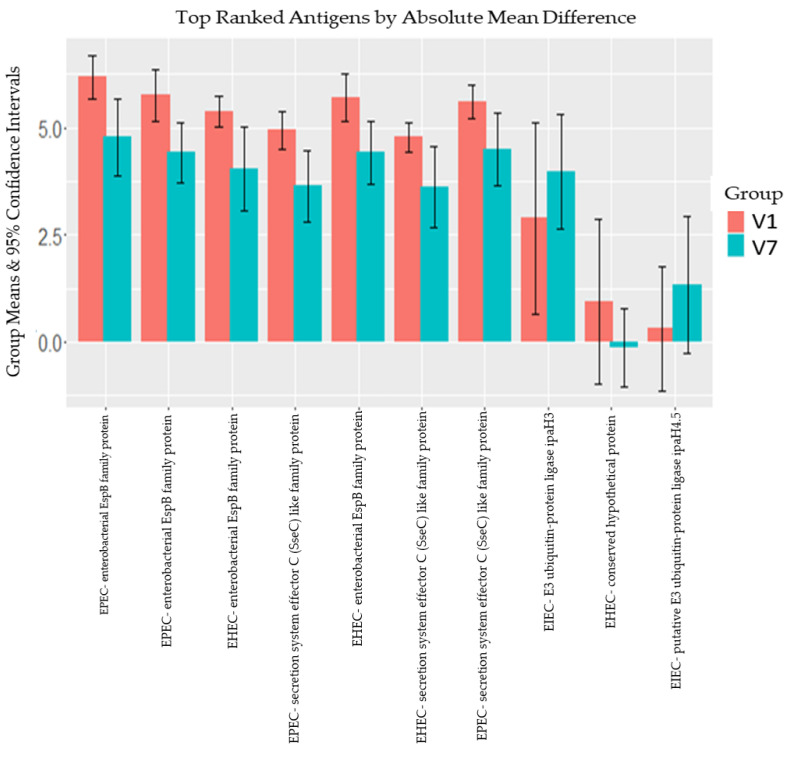
Antibody mean difference in placebo group between V1 (baseline) and V7 (7 days post third dose of placebo). The vertical error bars define the 95% confidence interval around each mean. The red bars represent the group mean signal intensities obtained at baseline (V1) while the blue bars represent the group mean signal intensities obtained 7 days post the administration of placebo (V7) for the third time.

The top 10 reactive proteins in the placebo group comprised those with the highest delta changes between V1 and V7 after averaging the signal intensities to all proteins at the two time points (Figure 3). As shown in Figure 3, the antibody responses to most of the top ten reactive proteins were lower at V7 than V1 in the placebo group except for two EIEC ipaH proteins (E3 ubiquitin–protein ligase ipaH3 and ipaH4.5).

### 3.5. Delta Changes in the Vaccinated and Placebo Group between V1 and V7

**Figure 4:**
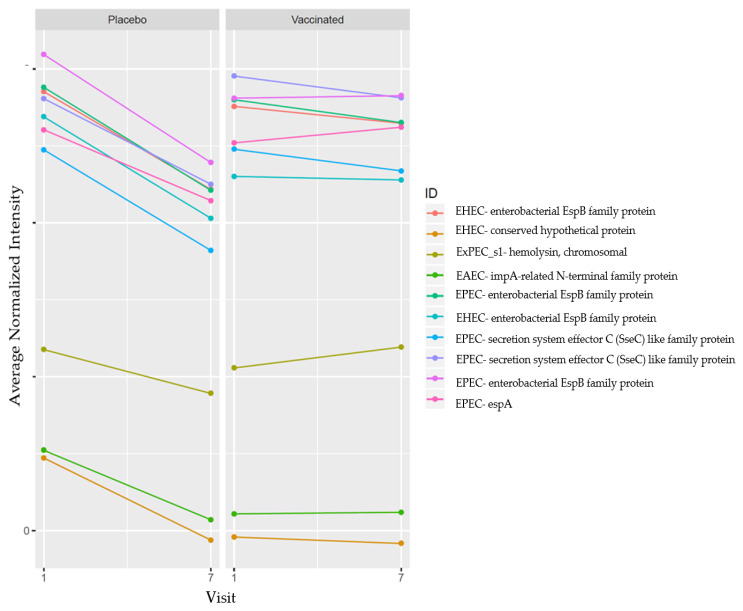
Antibody mean differences between V1 and V7 among both the vaccinated and placebo groups. The average intensity of reactivity to each protein was plotted at the two time points in order to visualize the delta changes. Each line represents a unique protein among the top 10 proteins with the highest delta changes in both groups.

Figure 4 shows the delta changes observed among the participants in both the vaccinated and placebo groups between V1 (the baseline) and V7 (seven days post the third dose). The proteins with the highest delta changes were selected for the visualization of the difference between placebo and vaccinated groups. There was a substantial decrease in reactivity intensity to all proteins among the placebo participants at V7. We noted a positive delta change to four proteins among vaccinated participants while the other proteins had a slight negative delta change. We observed a positive delta change to three DEC proteins in the vaccinated group (Figure 4).

### 3.6. Comparison of Mean IgG Reactivity Differences (V7-V1) between DEC and ETEC Proteins

**Figure 5:**
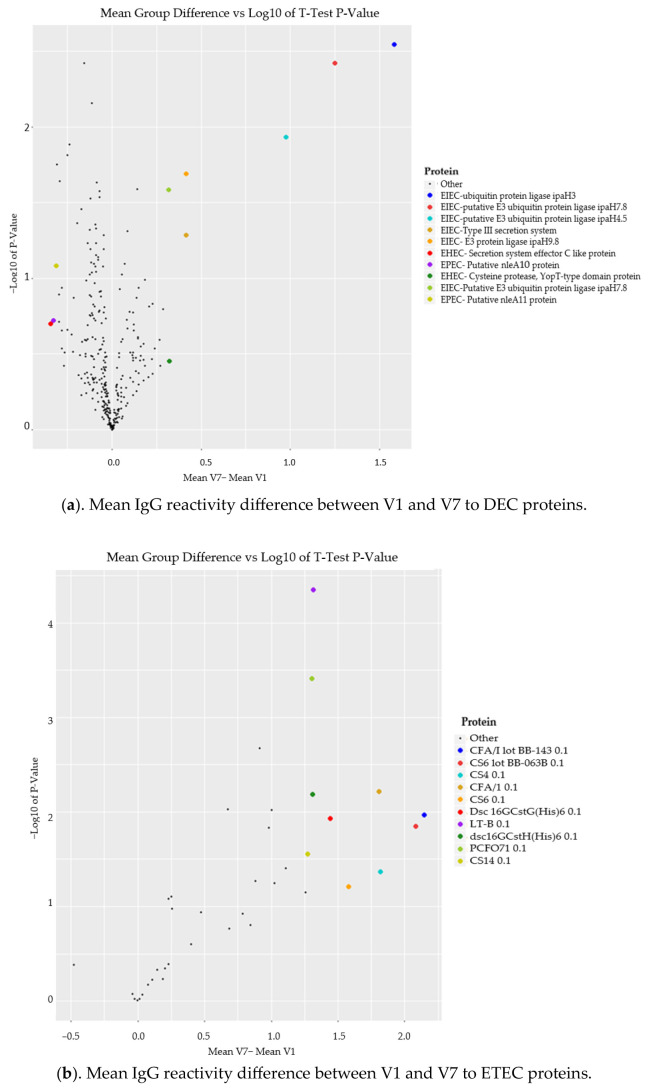
(**a**,**b**) Top 10 proteins by antibody reactivity mean. Antibody mean differences between V1 and V7 against DEC and ETEC proteins. The *x*-axis shows the difference in the group means (V1–V7), which was obtained by obtaining the average reactivity to each protein pre-vaccination and subtracting it from the average reactivity to each protein 7 days post the third dose of the vaccine. The *Y*-axis shows the log10 of the significance of the *t*-test *p*-value.

We compared the mean differences in reactivity within the vaccinated group between V1 and V7 in the DEC proteome versus the ETEC proteome from our previous work [26]. Our results in Figure 5a show that EIEC proteins were the top four DEC proteins by mean difference in the vaccinated group. Additionally, we observed that two out of the top ten of the DEC proteins had a difference greater than 1. In the ETEC proteome, we observed that in Figure 5b all top ten ETEC proteins were greater than 1. Two of these proteins, CFA/I and CS6 had a difference greater than 2 between V1 and V7. An increase of 1.0 signifies a 2-fold increase or a single doubling from V1 to V7. An increase of 2.0 signifies to a 4-fold increase or two doublings from V1 to V7.

## 4. Discussion

In this study, the top 20 reactive proteins were EPEC and EHEC derived across all the samples. Both pathogens belong to the attaching effacing family of enteric pathogens, whose pathogenesis is facilitated by the translocated intimin receptor (Tir) [31]. The high IgG reactivity to this protein (see Figure 1) suggests that it may have a very important function that the immune system attempts to prevent in order to stop the intimate attachment of the two pathogens, thus subverting the disease process as demonstrated in a previous study [32]. The elevated intensity of reactivity and positivity at baseline observed in all the samples suggests high and early exposure to these organisms among Zambian children possibly due to poor WASH [33]. These immunological observations drawn from the array data are consistent with the results of a recent epidemiological study assessing the burden of DEC among infants and young children in Zambia [8]. This implies that the vaccines may need to be administered earlier than 10 months to prevent infection among the children. Given that most bacterial vaccines may not be highly immunogenic among younger infants due to maternal interference, such a vaccine must be able to evade such interference to enhance its immunogenicity [34,35]. Of note also, we observed that there was an increased reactivity in the top 20 proteins among the vaccinated group as compared to the placebo.

Within the vaccinated group, we observed strong reactivity to EIEC-specific proteins post-vaccination. Being a region with a high *Shigella* burden, we attributed the high reactivity to natural infection in the children by *Shigella* spp. [36]. Although natural infection is a possible explanation, the IgG reactivity observed in the vaccinated group could also be attributable to the ETVAX^®^ vaccine as evidenced by the significantly higher readings post the last dose compared to pre-vaccination. Previous studies have reported cross-reactivity of antibodies against organisms of the same genus or species [24,37]. In addition, ETEC and EPEC have been shown to share specific virulence factors as well [12]. The plausible immunological basis of these observations is the conservation of epitope regions in organisms of the same species [23]. Furthermore, the ETVAX^®^ vaccine is made up of whole-cell bacteria implying that other uncontrolled proteins may trigger a response to organisms of the same species [18]. This phenomenon could be important in broadening vaccine coverage and improving the full public health value proposition for vaccination against these organisms in high disease-burden regions. Most of the EIEC proteins to which we observed high reactivity were invasion plasmid antigen (Ipa) proteins that are involved in the invasion of cells, which is a very important aspect of the virulence mechanism of this pathogen [38]. Although not statistically significant (*p*-value > 0.05), we observed that the reactivity was higher post-vaccination. This trend was observed in all the top 10 reactive proteins except one EHEC secretion system effector C (SseC) and two EPEC nleA proteins. The decline in reactivity to these proteins can be attributed to prior exposure at baseline and consequent waning over time suggesting that there was no boost of IgG response by the vaccine. The SseC proteins play a role in the adhesion of EHEC and nleA is involved in disrupting the tight junctions in EPEC infection [39,40]. Due to the crucial role that these proteins play in the pathogenesis of these pathogens, there is a need to further investigate them in future DEC vaccine work.

The top reactive proteins in the placebo group provided insight into the natural immune response to wild-type infection in the community. These proteins were the EspB and secretion system effector C (SseC) like family proteins derived from EHEC and EPEC pathotypes. They are both effector proteins that play a key role in the intimate adhesion of the organisms to the gut epithelium [39]. The EspB protein has also been reported to induce cell death in immune cells [41]. A previous study demonstrated that antibody binding to EspB protein significantly reduced the adhesion of EPEC [42]. This suggests that EspB could potentially be a good vaccine antigen target for the prevention of EPEC or EHEC adhesion. It has also been suggested as an antigen for inclusion in ETEC vaccines to improve coverage [6]. Preventing the adhesion of a pathogen substantially subverts the disease process as this is a crucial step in the pathogenesis of many organisms.

During the study, there was a larger decline in the intensity of reactivity among the placebo group compared to the vaccinated group between samples collected at baseline and those collected 7 days post the third dose of vaccination. We hypothesize that, despite not boosting the response to some proteins, the ETVAX^®^ vaccine may have kept the IgG antibody titers higher, thus offering some level of protection to EPEC, EHEC and EAEC proteins. The difference in the delta changes between the two groups is suggestive that the vaccine may have an effect on the reactivity of the proteins in our study. Observations from this pilot study are preliminary and require further work to build upon this work. Future research should consider employing a larger sample size and conducting active testing for DEC pathogens during the course of the study. This approach will aid investigators in definitively associating the heightened reactivity with the vaccine, thereby demonstrating cross-reactivity.

A further comparison of the mean differences in reactivity between the DEC (non-ETEC) and ETEC proteome from our previous work [26] revealed a superior intensity of reactivity to ETEC proteins. We noted that the proteins with the highest mean differences are part of the ETVAX vaccine [22], showing that protection against ETEC is more pronounced. Furthermore, we observed that all ETEC proteins had a positive change in reactivity while the DEC proteins had some reductions in reactivity post-full vaccination. This further affirms the superiority of the vaccine’s protective effect against ETEC.

Our study provides great insights into the high exposure of children in an LMIC setting to DEC and the possible cross-protection that the ETVAX^®^ vaccine may provide against other DEC. The small number of samples used in our study limits the extent to which we can conclusively attribute our observations to the ETVAX^®^ vaccine. However, this study illustrates the utility of the microarray as a tool in the field evaluations of exposure to multiple antigens. Several other important immune factors such as the IgA and secretory immunoglobulin were not evaluated in our study. Furthermore, our microarray did not include proteins obtained from hybrid or new emerging DEC strains. This means that the true impact of the ETVAX^®^ vaccine in protecting against other DECs may not have been fully explored. Our findings were not correlated to disease or its severity and, therefore, they must not be interpreted as clinically relevant observations at this stage. Future studies may need to investigate the effect of the ETVAX^®^ vaccine using functional assays such as bactericidal assays.

In conclusion, our study highlights the utility of proteomic array analysis of the proteomic microarray in supporting vaccine development and epidemiological studies seeking to better define the range of enteric pathogens contributing to the global enteric disease burden. We demonstrated for the first time that using the newly developed pan-DEC array ETVAX^®^ vaccine-induced antibodies may be cross-reactive to proteins derived from the EIEC, EHEC and EPEC pathotypes. An important note is that the proteins with the highest IgG reactivity in our study are known to have a critical role in the pathogenesis of these pathogens. We recommend future studies be designed to explore the usefulness of the proteins identified here as putative vaccine targets.

## Data Availability

The data are available in the NCBI Gene Expression Omnibus (GEO) repository. The GEO series accession number is GSE220814.

## Supplementary Materials

The following supporting information can be downloaded at: https://www.mdpi.com/article/10.3390/microorganisms12030420/s1, Table S1: Participant characteristics and Table S2: DEC proteins in the microarray.

## References

[1] Walker C.L.F., Rudan I., Liu L., Nair H., Theodoratou E., Bhutta Z.A., O’Brien K.L., Campbell H., Black R.E. (2013). Global Burden of Childhood Pneumonia and Diarrhoea. Lancet.

[2] Kotloff K.L., Nataro J.P., Blackwelder W.C., Nasrin D., Farag T.H., Panchalingam S., Wu Y., Sow S.O., Sur D., Breiman R.F. (2013). Burden and Aetiology of Diarrhoeal Disease in Infants and Young Children in Developing Countries (the Global Enteric Multicenter Study, GEMS): A Prospective, Case-Control Study. Lancet.

[3] Pinkerton R., Oriá R.B., Lima A.A.M., Rogawski E.T., Oriá M.O.B., Patrick P.D., Moore S.R., Wiseman B.L., Niehaus M.D., Guerrant R.L. (2016). Early Childhood Diarrhea Predicts Cognitive Delays in Later Childhood Independently of Malnutrition. Am. J. Trop. Med. Hyg..

[4] Osawa K., Raharjo D., Wasito E.B., Harijono S., Shigemura K., Osawa R., Sudarmo S.M., Iijima Y., Shirakawa T. (2013). Frequency of Diarrheagenic *Escherichia coli* among Children in Surabaya, Indonesia. Jpn. J. Infect. Dis..

[5] Pérez C., Gómez-Duarte O.G., Arias M.L. (2010). Diarrheagenic *Escherichia coli* in Children from Costa Rica. Am. J. Trop. Med. Hyg..

[6] Khalil I., Walker R., Porter C.K., Muhib F., Chilengi R., Cravioto A., Guerrant R., Svennerholm A.-M., Qadri F., Baqar S. (2021). Enterotoxigenic *Escherichia coli* (ETEC) Vaccines: Priority Activities to Enable Product Development, Licensure, and Global Access. Vaccine.

[7] Geurtsen J., de Been M., Weerdenburg E., Zomer A., McNally A., Poolman J. (2022). Genomics and Pathotypes of the Many Faces of *Escherichia coli*. FEMS Microbiol. Rev..

[8] Mwape K., Bosomprah S., Chibesa K., Silwamba S., Luchen C.C., Sukwa N., Mubanga C., Phiri B., Chibuye M., Liswaniso F. (2023). Prevalence of Diarrhoeagenic *Escherichia coli* among Children Aged between 0–36 Months in Peri-Urban Areas of Lusaka. Microorganisms.

[9] Gomes T.A.T., Elias W.P., Scaletsky I.C.A., Guth B.E.C., Rodrigues J.F., Piazza R.M.F., Ferreira L.C.S., Martinez M.B. (2016). Diarrheagenic *Escherichia coli*. Braz. J. Microbiol. Publ. Braz. Soc. Microbiol..

[10] Yu F., Chen X., Zheng S., Han D., Wang Y., Wang R., Wang B., Chen Y. (2018). Prevalence and Genetic Diversity of Human Diarrheagenic *Escherichia coli* Isolates by Multilocus Sequence Typing. Int. J. Infect. Dis..

[11] Thakur N., Jain S., Changotra H., Shrivastava R., Kumar Y., Grover N., Vashistt J. (2018). Molecular Characterization of Diarrheagenic *Escherichia coli* Pathotypes: Association of Virulent Genes, Serogroups, and Antibiotic Resistance among Moderate-to-Severe Diarrhea Patients. J. Clin. Lab. Anal..

[12] Hazen T.H., Michalski J., Luo Q., Shetty A.C., Daugherty S.C., Fleckenstein J.M., Rasko D.A. (2017). Comparative Genomics and Transcriptomics of *Escherichia coli* Isolates Carrying Virulence Factors of Both Enteropathogenic and Enterotoxigenic *E. coli*. Sci. Rep..

[13] Taylor D.N., Bourgeois A.L., Ericsson C.D., Steffen R., Jiang Z.-D., Halpern J., Haake R., Dupont H.L. (2006). A Randomized, Double-Blind, Multicenter Study of Rifaximin Compared with Placebo and with Ciprofloxacin in the Treatment of Travelers’ Diarrhea. Am. J. Trop. Med. Hyg..

[14] Okeke I.N., Aboderin O.A., Byarugaba D.K., Ojo K.K., Opintan J.A. (2007). Growing Problem of Multidrug-Resistant Enteric Pathogens in Africa. Emerg. Infect. Dis..

[15] Ingle D.J., Levine M.M., Kotloff K.L., Holt K.E., Robins-Browne R.M. (2018). Dynamics of Antimicrobial Resistance in Intestinal *Escherichia coli* from Children in Community Settings in South Asia and Sub-Saharan Africa. Nat. Microbiol..

[16] Msolo L., Iweriebor B.C., Okoh A.I. (2020). Antimicrobial Resistance Profiles of Diarrheagenic *E. coli* (DEC) and Salmonella Species Recovered from Diarrheal Patients in Selected Rural Communities of the Amathole District Municipality, Eastern Cape Province, South Africa. Infect. Drug Resist..

[17] Ohwo O., Agusomu T.D. (2018). Assessment of Water, Sanitation and Hygiene Services in Sub-Saharan Africa. Eur. Sci. J. ESJ.

[18] Qadri F., Akhtar M., Bhuiyan T.R., Chowdhury M.I., Ahmed T., Rafique T.A., Khan A., Rahman S.I.A., Khanam F., Lundgren A. (2020). Safety and Immunogenicity of the Oral, Inactivated, Enterotoxigenic *Escherichia coli* Vaccine ETVAX in Bangladeshi Children and Infants: A Double-Blind, Randomised, Placebo-Controlled Phase 1/2 Trial. Lancet Infect. Dis..

[19] Darsley M.J., Chakraborty S., DeNearing B., Sack D.A., Feller A., Buchwaldt C., Bourgeois A.L., Walker R., Harro C.D. (2012). The Oral, Live Attenuated Enterotoxigenic *Escherichia coli* Vaccine ACE527 Reduces the Incidence and Severity of Diarrhea in a Human Challenge Model of Diarrheal Disease. Clin. Vaccine Immunol. CVI.

[20] Riddle M.S., Maciel M.J., Porter C.K., Poole S.T., Gutierrez R.L., Gormley R., Laird R.M., Sebeny P.J., Dori K.E., Greenleaf M.E. (2020). A First in Human Clinical Trial Assessing the Safety and Immunogenicity of Transcutaneously Delivered Enterotoxigenic *Escherichia coli* Fimbrial Tip Adhesin with Heat-Labile Enterotoxin with Mutation R192G. Vaccine.

[21] Fleckenstein J.M. (2021). Confronting Challenges to Enterotoxigenic *Escherichia coli* Vaccine Development. Front. Trop. Dis..

[22] Holmgren J., Bourgeois L., Carlin N., Clements J., Gustafsson B., Lundgren A., Nygren E., Tobias J., Walker R., Svennerholm A.-M. (2013). Development and Preclinical Evaluation of Safety and Immunogenicity of an Oral ETEC Vaccine Containing Inactivated *E. coli* Bacteria Overexpressing Colonization Factors CFA/I, CS3, CS5 and CS6 Combined with a Hybrid LT/CT B Subunit Antigen, Administered Alone and Together with dmLT Adjuvant. Vaccine.

[23] Vojtek I., Buchy P., Doherty T.M., Hoet B. (2019). Would Immunization Be the Same without Cross-Reactivity?. Vaccine.

[24] Shekhar S., Khan R., Ferreira D.M., Mitsi E., German E., Rørvik G.H., Berild D., Schenck K., Kwon K., Petersen F. (2018). Antibodies Reactive to Commensal *Streptococcus mitis* Show Cross-Reactivity with Virulent *Streptococcus pneumoniae* Serotypes. Front. Immunol..

[25] Ndungo E., Randall A., Hazen T.H., Kania D.A., Trappl-Kimmons K., Liang X., Barry E.M., Kotloff K.L., Chakraborty S., Mani S. (2018). A Novel Shigella Proteome Microarray Discriminates Targets of Human Antibody Reactivity Following Oral Vaccination and Experimental Challenge. mSphere.

[26] Mubanga C., Simuyandi M., Mwape K., Chibesa K., Chisenga C., Chilyabanyama O.N., Randall A., Liang X., Glashoff R.H., Chilengi R. (2023). Use of an ETEC Proteome Microarray to Evaluate Cross-Reactivity of ETVAX^®^ Vaccine-Induced IgG Antibodies in Zambian Children. Vaccines.

[27] Prechl J., Papp K., Erdei A. (2010). Antigen Microarrays: Descriptive Chemistry or Functional Immunomics?. Trends Immunol..

[28] Sukwa N., Mubanga C., Hatyoka L.M., Chilyabanyama O.N., Chibuye M., Mundia S., Munyinda M., Kamuti E., Siyambango M., Badiozzaman S. (2023). Safety, Tolerability, and Immunogenicity of an Oral Inactivated ETEC Vaccine (ETVAX^®^) with dmLT Adjuvant in Healthy Adults and Children in Zambia: An Age Descending Randomised, Placebo-Controlled Trial. Vaccine.

[29] Chakraborty S., Randall A., Vickers T.J., Molina D., Harro C.D., DeNearing B., Brubaker J., Sack D.A., Bourgeois A.L., Felgner P.L. (2018). Human Experimental Challenge with Enterotoxigenic *Escherichia coli* Elicits Immune Responses to Canonical and Novel Antigens Relevant to Vaccine Development. J. Infect. Dis..

[30] Chakraborty S., Randall A., Vickers T.J., Molina D., Harro C.D., DeNearing B., Brubaker J., Sack D.A., Bourgeois A.L., Felgner P.L. (2019). Interrogation of a Live-Attenuated Enterotoxigenic *Escherichia coli* Vaccine Highlights Features Unique to Wild-Type Infection. NPJ Vaccines.

[31] Mühlen S., Zapol’skii V.A., Bilitewski U., Dersch P. (2021). Identification of Translocation Inhibitors Targeting the Type III Secretion System of Enteropathogenic *Escherichia coli*. Antimicrob. Agents Chemother..

[32] Kordbacheh E., Nazarian S., Hajizadeh A., Fasihi-Ramandi M., Fathi J. (2019). Recombinant HcpA-EspA-Tir-Stx2B Chimeric Protein Induces Immunity against Attachment and Toxicity of *Escherichia coli* O157:H7. Microb. Pathog..

[33] Nyambe S., Agestika L., Yamauchi T. (2020). The Improved and the Unimproved: Factors Influencing Sanitation and Diarrhoea in a Peri-Urban Settlement of Lusaka, Zambia. PLoS ONE.

[34] Giugliano L.G., Ribeiro S.T., Vainstein M.H., Ulhoa C.J. (1995). Free Secretory Component and Lactoferrin of Human Milk Inhibit the Adhesion of Enterotoxigenic *Escherichia coli*. J. Med. Microbiol..

[35] Brandtzaeg P. (2010). The Mucosal Immune System and Its Integration with the Mammary Glands. J. Pediatr..

[36] Miti S., Chilyabanyama O.N., Chisenga C.C., Chibuye M., Bosomprah S., Mumba C., Chitondo S., Siziya S., Cohen D., Chilengi R. (2023). Sensitivity and Predictive Value of Dysentery in Diagnosing Shigellosis among under Five Children in Zambia. PLoS ONE.

[37] Calderon Toledo C., Arvidsson I., Karpman D. (2011). Cross-Reactive Protection against Enterohemorrhagic *Escherichia coli* Infection by Enteropathogenic *E. coli* in a Mouse Model. Infect. Immun..

[38] Yang S.-C., Hung C.-F., Aljuffali I.A., Fang J.-Y. (2015). The Roles of the Virulence Factor IpaB in *Shigella* spp. in the Escape from Immune Cells and Invasion of Epithelial Cells. Microbiol. Res..

[39] Gaytán M.O., Martínez-Santos V.I., Soto E., González-Pedrajo B. (2016). Type Three Secretion System in Attaching and Effacing Pathogens. Front. Cell. Infect. Microbiol..

[40] Thanabalasuriar A., Koutsouris A., Weflen A., Mimee M., Hecht G., Gruenheid S. (2010). The Bacterial Virulence Factor NleA Is Required for the Disruption of Intestinal Tight Junctions by Enteropathogenic *Escherichia coli*. Cell. Microbiol..

[41] Baumann D., Salia H., Greune L., Norkowski S., Körner B., Uckeley Z.M., Frankel G., Guenot M., Rüter C., Schmidt M.A. (2018). Multitalented EspB of Enteropathogenic *Escherichia coli* (EPEC) Enters Cells Autonomously and Induces Programmed Cell Death in Human Monocytic THP-1 Cells. Int. J. Med. Microbiol. IJMM.

[42] Li D., Chen Z., Cheng H., Zheng J.-X., Pan W.-G., Yang W.-Z., Yu Z.-J., Deng Q.-W. (2016). Inhibition of Adhesion of Enteropathogenic *Escherichia coli* to HEp-2 Cells by Binding of a Novel Peptide to EspB Protein. Curr. Microbiol..

