# Supplementary material for: Application of a Novel Proteomic Microarray Reveals High Exposure to Diarrhoeagenic Escherichia coli among Children in Zambia Participating in a Phase I Clinical Trial
Source: Microorganisms. 2024 Feb 20;12(3):420. doi: 10.3390/microorganisms12030420 (PMC10972023; doi:10.3390/microorganisms12030420)
Supplement: Supplementary file 1 [file microorganisms-12-00420-s001.zip › Supplementary table S1-Participant characteristics.pdf]

Table s1. Participant characteristics

Study ID	Age	Gender	Sick visit
**Placebo**			
S078-2010	20	Male	Acute gastroenteritis
S070-2013	23	Female	None
S087-2019	22	Female	None
S129-2043	23	Female	None
**1/8th Dose**			
S060-2002	18	Female	None
S058-2007	15	Male	Acute diarrhoeal disease
S095-2025	19	Female	None
S103-2031	14	Female	Acute gastroenteritis
S107-2037	21	Female	Acute gastroenteritis
S147-2049	21	Female	None
S148-2055	17	Female	Acute gastroenteritis
S142-2057	16	Male	Acute gastroenteritis
**1/4 Dose**			
S064-2004	13	Male	None
S085-2016	18	Male	None
S094-2022	14	Male	None
S104-2028	21	Female	None
S114-2034	23	Female	None
S125-2040	17	Female	None
S131-2046	18	Female	Acute gastroenteritis
S145-2052	20	Female	None
